# Comparative Transcriptomic Analysis of Three Common Liver Cell Lines

**DOI:** 10.3390/ijms24108791

**Published:** 2023-05-15

**Authors:** Viktoriia Arzumanian, Mikhail Pyatnitskiy, Ekaterina Poverennaya

**Affiliations:** Institute of Biomedical Chemistry, 119121 Moscow, Russia; arzumanian.victoria@gmail.com (V.A.);

**Keywords:** liver cell lines, HepG2, Huh7, Hep3B, RNAseq

## Abstract

Background: Comparative transcriptomic analysis is a powerful approach for investigating the molecular mechanisms underlying various physiological and pathological processes, including liver disease. The liver is a vital organ with diverse functions, including metabolism and detoxification. In vitro models of liver cells, such as HepG2, Huh7, and Hep3B, have been widely used to study liver biology and pathology. However, there is limited information on the heterogeneity of these cell lines at the transcriptomic level. Objective: This study aimed to conduct a comparative transcriptomic analysis of three common liver cell lines (HepG2, Huh7, and Hep3B) using publicly available RNA-sequencing data. In addition, we compared these cell lines to primary hepatocytes, cells isolated directly from liver tissue and considered the gold standard for studying liver function and disease. Methods: Our study included sequencing data with the following criteria: total number of reads over 20,000,000, average read length of over 60 base pairs, Illumina sequencing, and non-treated cells. The data for the three cell lines were compiled: HepG2 (97 samples), Huh7 (39 samples), and Hep3B (16 samples). We performed differential gene expression analysis using the DESeq2 package, principal component analysis, hierarchical clustering on principal components, and correlation analysis to explore the heterogeneity within each cell line. Results: We identified numerous genes and pathways differentially expressed between HepG2, Huh7, and Hep3B, such as oxidative phosphorylation, cholesterol metabolism, and DNA damage. We report that the expression levels of important genes differ significantly between primary hepatocytes and liver cell lines. Conclusion: Our study provides new insights into the transcriptional heterogeneity of commonly used liver cell lines and highlights the importance of considering specific cell line. Consequently, transferring results without considering the heterogeneity of cell lines is impractical and may lead to inaccurate or distorted conclusions.

## 1. Introduction

Human cancer cell lines have been extensively employed as in vitro models for cancer research and drug development. Cell lines can be used to study cell signaling, gene expression, and cell differentiation. For research outcomes to be reliably reproduced between laboratories, it is ideal that these cell lines exhibit genetic stability and cellular profiles. Unfortunately, several studies have highlighted various issues in human cancer cell lines: cell line misidentification, cross-contamination, and poor annotation that could impair the reproducibility of results obtained from these cell lines between laboratories. To authenticate cells, the researchers proposed short tandem repeat and single nucleotide polymorphism profiles. However, genetic changes during culturing and passage can affect gene expression and eventually cellular functions. In 2018, Ben-David et al. investigated 27 strains of the breast cancer cell line MCF7 and reported genetic heterogeneity and widely varied drug responses to 321 anti-cancer compounds [1]. Other researchers also showed that HeLa cells differ significantly in genomic and transcriptome profiles between 13 laboratories [2].

Immortalized liver cell lines are more commonly used in research than liver biopsies because of their low cost, ease of use, and retention of most key enzyme activities. To date, more than 40 hepatic lines, including HepaRG, Huh7, SK-Hep-1, Hep3B, and HepG2, are frequently used [3]. These cell lines are considered neoplastic and are models for three cancers, such as hepatocellular carcinoma (HepaRG, Hep3B, and Huh7), hepatoblastoma (HepG2), and adenocarcinoma (SK-Hep-1). All five lines have different number of chromosomes and mutational profile [4].

Understanding the degree of heterogeneity within and between different liver cell lines is of utmost importance for researchers using these cells as a model system to study various biological processes, including drug metabolism, gene regulation, and liver disease. Despite their widespread use, the heterogeneity of liver cell lines can present challenges that may impact the reproducibility and interpretability of experimental findings.

In this study, we performed a comparative transcriptomic analysis of three commonly used liver cell lines: HepG2, Huh7, and Hep3B. These cell lines were selected due to their popularity in research and the availability of publicly accessible RNA sequencing data. We aimed to explore the heterogeneity within each cell line and compare their transcriptomic profiles with each other. To achieve these goals, we used principal component analysis (PCA), hierarchical component clustering (HCPC), and correlation analysis to identify gene expression patterns that distinguished the cell lines from each other and within samples. Additionally, we utilized the DESeq2 package [5] to identify differentially expressed genes. Our findings underscore the biological intricacies of commonly utilized liver cell lines and establish a vital foundation for debating how experimental data derived from these cell lines should be presented and comprehended.

## 2. Results

In order to investigate the heterogeneity of liver cell lines, we conducted a comprehensive analysis using publicly available data for HepG2, Huh7, and Hep3B cell lines downloaded from the NCBI SRA database. In addition, we incorporated our own data for HepG2 cells sequenced using Illumina NovaSeq 6000 with a read length of 100 base pairs (bp). We focused on control samples that represented the baseline cell lines in their natural, untreated state. This data is fundamental to comprehending cells and is crucial for discerning and evaluating any alterations that may transpire due to treatments, storage, or other influences.

**Figure 1:**
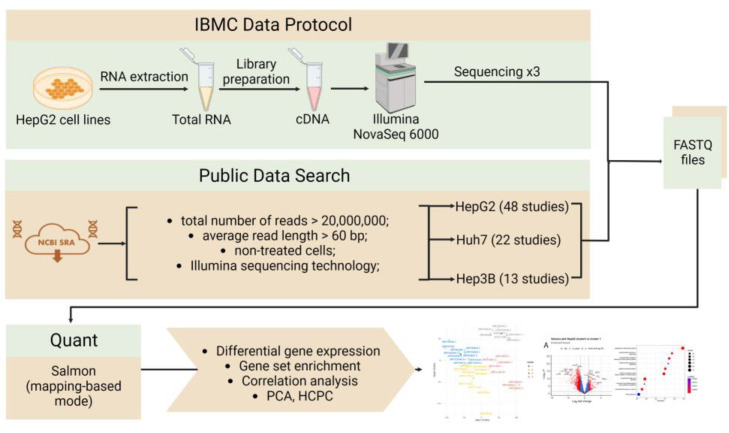
The overall workflow of the experiment comparing the popular liver cell lines.

The data processing pipeline consisted of several key steps, including quality control, read trimming, alignment to the reference transcriptome, and quantification of gene expression. We performed the final step of the analysis in R, utilizing various statistical and bioinformatics tools such as the PCA, correlation analysis, differential expression (DESeq2 package), and functional enrichment analysis. The overall workflow of the experiment and a summary of data processing are presented in Figure 1.

As a result of two transcriptomic profilings at the Institute of Biomedical Chemistry (IBMC), expression data were obtained for the HepG2 cell line in different years (Supplementary File S1, Table S1). The first batch of IBMC data (IBMC.1) is described in Pyatnitskiy et al. [6], and the raw data is available from the NCBI SRA repository under study accession number PRJNA765908. The second batch of IBMC data (IBMC.2) was obtained in 2021 (see Section 4.1, Section 4.2 and Section 4.3 above), and the raw data is available from the NCBI SRA repository under study accession number PRJNA956723. It should be noted that the HepG2 cell line is certified and was obtained directly from the Sigma–Aldrich biobank (passage 5).

After performing the quality control of the IBMC data, we defined criteria for published data from the Huh7, Hep3B, and HepG2 cell lines to be comparable in quality. The criteria were as follows: total number of reads >20,000,000; average read length >60 bp; non-treated cells; Illumina sequencing technology.

Despite the fact that more than 10,000 RNAseq samples are available for liver cell lines in the NCBI SRA database, the total number of obtained samples matching our criteria makes it possible to compare only three cell lines—HepG2, Hep3B, and Huh7 (Supplementary File S1, Figure S1). For the HepG2 cell line, total 8406 results were found using search parameters: “HepG2”[All Fields] AND “rna seq”[Strategy] AND (“biomol rna”[Properties] AND “platform illumina”[Properties] AND “filetype fastq”[Properties]). To reduce the results to control data only, we add an additional parameter: “control” [All fields]. This reduced the results to 634, of which 97 samples were selected according to the above criteria. Using similar search parameters for Huh7 cells, we found 3601 and 189 results, respectively. We found 39 samples for Huh7 cell lines. For the Hep3B cell line, out of 219 results, only 39 remained, of which 16 samples satisfied our criteria. Accession numbers are listed in Supplementary Table S2.

A quality check of the selected public data revealed that, on average, the number of fragments in cell line samples was 31 million reads, and the average read length was 128 bp. In contrast, the length of reads in the IBMC data averaged 100 bp, and the number of reads exceeded the average value in the published data and equaled 69 million. Information regarding the average data quality for each cell line is presented in Table 1, while more details for each sample are available in Supplementary Table S2.

### 2.1. Comparison of Cell Lines between Study

As cell lines vary between laboratories, we performed statistical analyses to assess potential “laboratory effects”. This involved conducting correlation and PCA analyses and examining differentially expressed genes between the resulting clusters.

#### 2.1.1. Comparison of HepG2 Cell Line between Studies

Correlation analysis indicated a strong positive correlation between the expression levels of transcripts and genes in the HepG2 cell line samples. The average Spearman correlation coefficient between samples was 0.70 at the transcript level and more than 0.86 at the gene level (Supplementary File S1, Figure S2). This suggests a high similarity in the transcriptome profiles of the HepG2 samples.

Additionally, to identify the distribution of samples in the HepG2 dataset, we performed hierarchical clustering on principal components (HCPC) analysis. In this approach, gene expression data are first pre-processed and normalized, followed by the calculation of the principal components representing the main sources of variation in the data. A hierarchical clustering algorithm was then applied to the principal components.

**Figure 2:**
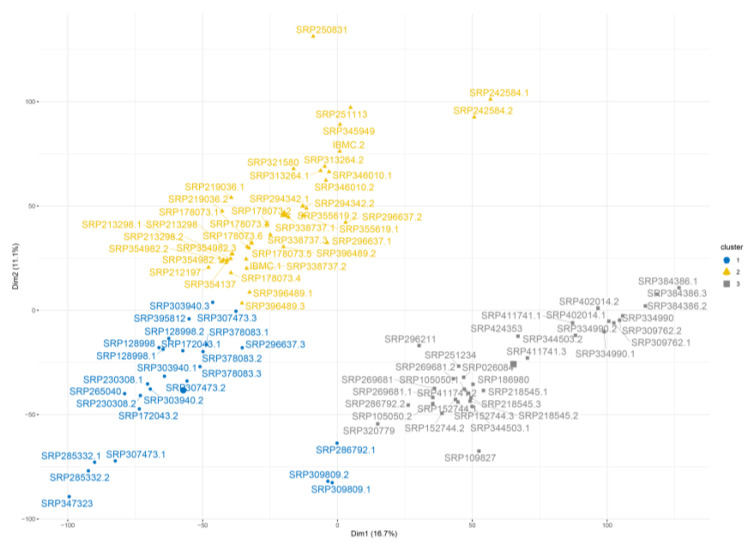
HCPC analysis on genes level for HepG2 cell line.

The analysis revealed that the HepG2 cell line data were divided into three clusters based on their transcript (Supplementary File S1, Figure S3) and gene levels (Figure 2). The second cluster included 49 samples (represented in yellow), which included IBMC data (IBMC.1 and IBMC.2). Clusters #1 and #3 contain 25 and 29 samples, respectively.

Finally, we used differential gene expression analysis to identify genes that were differentially expressed in the third cluster identified by HCPC analysis. The comparison results for each cluster pair are presented in Table 2 and Supplementary Table S3, and Supplementary File S1 (Figure S4) shows the volcano and enrichment plots. We found the most significant number of differentially expressed genes (2871) between clusters #3 and #1.

The pathways that show differences between the clusters #1 and #2 in HepG2 cells are essential for energy production and proper mitochondrial function. These pathways include “The Electron Transport Chain (ETC) Oxidative Phosphorylation (OXPHOS) system in mitochondria” (FDR = 1.58 × $10^{-8}$), “The Oxidative Phosphorylation” (FDR = 1.22 × $10^{-6}$), and the assembly of mitochondrial complexes I (FDR = 5.07 × $10^{-6}$), III (FDR < 0.001) and IV (FDR = 1.84 × $10^{-5}$). They all work together to generate ATP via OXPHOS, a crucial source of cellular energy in cells, and maintain the proper function of the mitochondria, which is essential for normal metabolism, biosynthesis, and the survival of the cells [7]. Changes in the pathway of the electron transport chain OXPHOS system in the mitochondria (FDR = 0.04) were also observed between clusters #1 and #3.

“The cytoplasmic ribosomal proteins” (FDR = 1.58 × $10^{-8}$), “The retinoblastoma gene” (FDR = 3.79 × $10^{-7}$), and “The focal adhesion” (FDR < 0.001) pathways were also common to the results in the analysis of clusters #1 vs #2 and #1 vs #3. Cytoplasmic ribosomal proteins are important for the formation of large and small ribosomal subunits, key ribosome components, and the cellular machinery responsible for protein synthesis. Thus, changes in cytoplasmic ribosomal protein expression in HepG2 cells could potentially affect the Wnt pathway and subsequently impact cell function. In addition, “The retinoblastoma (RB) gene in cancer” (RB) pathway regulates cell cycle progression and proliferation of HepG2 cells [8]. Mutations or alterations in the RB1 gene can lead to cancer development, including liver cancer. A member of this pathway, the *RRM1* gene, is up-regulated in cluster #2 (log2fc = 2.07, FDR = 6.25 × $10^{-8}$) compared to cluster #1. In HepG2 cells, dysregulation of *RRM1* may contribute to the progression of liver cancer by promoting DNA synthesis and cell proliferation. The focal adhesion pathway also differed between clusters #1 and #2. Dysregulation of the focal adhesion pathway in HepG2 cells can lead to aberrant cell adhesion, migration, and invasion, all of which are critical for cancer progression and metastasis.

Changes were also observed between clusters #1 and #2 in the pathway associated with “The non-alcoholic fatty liver disease (NAFLD)” (FDR = 1.58 × $10^{-8}$). This pathway participates in lipid uptake, synthesis, and storage in the liver and the regulation of inflammation and oxidative stress. Changes in this pathway can lead to more severe forms of liver disease, such as non-alcoholic steatohepatitis and liver fibrosis.

As a result of comparing clusters #1 and #3, we observed differences in the pathways—“The oxidation by cytochrome P450” (FDR = 0.01) and “Metapathway biotransformation phase I and II” (FDR < 0.001). The stability of these pathways is vital for cell lines because the liver is the main site of drug metabolism, and HepG2 cells are commonly used as a model system for studying the metabolism and toxicity of drugs and other xenobiotics [4]. Genes *CYP*, *UGT*, *SULT*, and *GST* are primarily involved in drug metabolism and detoxification. We also observed changes in “The biotransformation phase I and II metapathway” (FDR < 0.001) between clusters #2 and #3. Differential expression analysis showed that in the #3 cluster, compared to the #1 and #2 clusters, there was a reduced expression of genes associated with pathways involved in drug metabolism and detoxification, such as *CYP3A5*, *CYP20A1*, *SULT1A4*, and *SULT1A2*.

Interestingly, in the analysis of cluster #1 vs #3 (FDR = 0.009) and #2 vs #3 (FDR = 0.03), changes in the pathway associated with Joubert syndrome were observed. Differential gene expression analysis showed increased *AHI1* and *TMEM67* expression in the cluster #3 compared with that in the #1 and #2 clusters. These genes are involved in ciliary assembly, maintenance, and signaling pathways, and mutations in these genes are known to cause Joubert syndrome.

The analysis of HepG2 cell line showed heterogeneity in several critical pathways, including the electron transport chain, oxidative phosphorylation, assembly of mitochondrial complexes I, IV, and III, cytoplasmic ribosomal proteins, retinoblastoma gene, and cytochrome P450 pathways. These pathways play a critical role in ATP production, maintenance of mitochondrial function, regulation of the cell cycle and proliferation, formation of ribosomal subunits, and drug metabolism. Changes in these pathways highlight the importance of careful transferring the results from one cell line to another.

#### 2.1.2. Comparison of Huh7 Cell Line between Studies

We observed a significant average Spearman coefficient between samples: at the transcript level, 0.7, and higher at the gene level, 0.85 (Supplementary File S1, Figure S5). The results indicated a high degree of similarity in transcriptome profiles between the different Huh7 samples.

**Figure 3:**
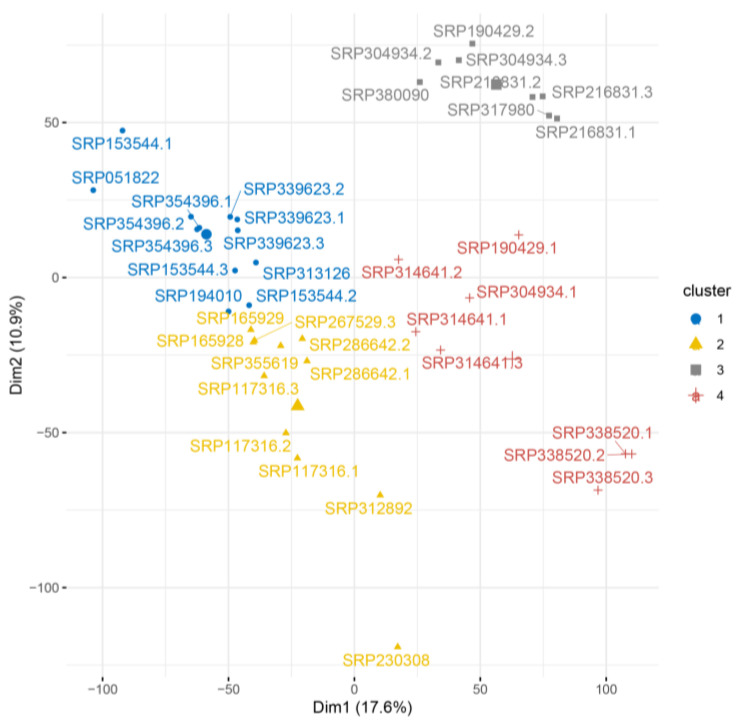
HCPC analysis at gene level for Huh7 cell line.

HCPC analysis showed that for the Huh7 cell line, five clusters were observed at the transcript level (Supplementary File S1, Figure S6). At the gene level, the number of clusters decreases to four (Figure 3). In both cases, most samples from the same study were in the same cluster, with some exceptions. The samples from the SRP304934 study were in different clusters, indicating internal heterogeneity. To understand how the samples differ from each other, we examined the value of the correlation between the data. The average Spearman correlation value between SRP304934.1 and SRP304934.2/3 was very high (0.96). The Spearman correlation between samples SRP304934.2 and SRP304934.3, which are in the same cluster is 0.98.

In order to examine the affected pathways between the four clusters in more detail, we performed a differential gene expression analysis. The comparison of the results for each pair of clusters is presented in Table 3 and Supplementary Table S4, and Supplementary File S1 (Figures S7 and S8) shows the volcano and enrichment plots. We found the most significant number of differentially expressed genes (3575) between clusters #3 and #1.

Between the clusters #1 and #2, there were changes in the DNA damage-related pathway—“DNA IR-damage and cellular response via ATR” (FDR = 0.009) as DNA damage sensors in Huh7 cells [9]. The gene encoding the ATR protein did not change significantly between clusters (log2fc = −0.7, FDR = 0.04). DNA damage can also lead to activation of the “Retinoblastoma (RB) gene in cancer pathway” (FDR = 0.015). The RB pathway is critical for regulating cell cycle progression and cell death. The components of this pathway, especially the *CDKN2A* gene encoding the p16Ink4a protein and RB, are often altered in sporadic human cancer, contributing to the dysregulation of cell proliferation [10]. The *RB1* gene, which encodes the RB protein, did not change between the clusters. At the same time, *CDKN2A* expression increased in cluster #2 compared to cluster #1 (log2fc =1.2, FDR = 0.004). The cytoplasmic ribosomal protein pathway (FDR = 0.02), which is responsible for cellular protein synthesis, is involved in ribosome production. DNA damage can potentially affect this pathway [11].

A pairwise comparison of clusters #4 and #3 with clusters #1 and #2 showed impairment in cholesterol metabolism and synthesis pathways: “Cholesterol metabolism with Bloch and Kandutsch–Russell pathways” and “Cholesterol biosynthesis pathway”. In addition, between clusters #4 and #2 and #4 and #1, the “Sterol regulatory element-binding proteins SREBP signaling” pathway is disrupted. All three pathways affect cholesterol metabolism and synthesis, which is one of the main sources of research on hepatic cell lines [12,13]. The key genes participating in these pathways’ change between clusters. Thus, in cluster #4, compared to cluster #2, genes *DHCR7* (log2fc = −1.12, FDR < 0.001), *DHCR24* (log2fc = −1.29, FDR = 2.47 × $10^{-6}$), *HMGCS1* (log2fc = −3.68, FDR = 3.59 × $10^{-19}$) and *SQLE* (log2fc = −1.09, FDR < 0.001) have been downregulated. The *HMGCS1* (log2fc = −3.98, FDR = 3.23 × $10^{-29}$) and *SQLE* (log2fc = −1.03, FDR = 1.62 × $10^{-6}$) genes also decreased in cluster #4 compared to cluster #1. In the cluster #3, compared to the cluster #2, the following genes decreased their expression: *DHCR7* (log2fc = −2.09, FDR = 4.11 × $10^{-10}$), *HMGCS1* (log2fc = −4.27, FDR = 3.32 × $10^{-27}$) and *DHCR24* (log2fc = −2.21, FDR = 3.23 × $10^{-25}$). Furthermore, reduced expression of these genes in the cluster #3 was observed compared to the cluster #1. We did not observe any changes in these genes between clusters #3 and #4.

The “Electron transport chain OXPHOS system in mitochondria” and “Mitochondrial complex I assembly model OXPHOS system” pathways associated with energy production in mitochondria were disrupted between the clusters. The disruption of these processes can lead to mitochondrial dysfunction and cell death. Thus, in the cluster #4, compared to the cluster #1 expression of the following genes was upregulated: *SDHA* (log2fc = 1.4, FDR = 1.4 × $10^{-6}$), *COX4I1* (log2fc = 1.08, FDR = 5.79 × $10^{-5}$), and *COX6A1* (log2fc = 1.74, FDR = 6.01 × $10^{-9}$). These are the crucial genes that participate in these pathways; additionally, part of these genes increased in cluster #4 compared to cluster #3, such as *COX4I1* (log2fc = 1.16, FDR = 4.65 × $10^{-6}$) and *COX6A1* (log2fc = 1.02, FDR = 0.0001).

In conclusion, our analysis revealed significant changes in various biological pathways in Huh7 cell clusters. The pathways associated with cholesterol metabolism, synthesis, and DNA damage are affected. In addition, changes in the pathways associated with mitochondrial energy production have been observed, which can lead to mitochondrial dysfunction and cell death. These results provide important information regarding the molecular mechanisms underlying the functional diversity of Huh7 cells and highlight the importance of accurately transferring results from one study to another.

#### 2.1.3. Comparison of Hep3B Cell Line between Studies

A strong and significant correlation was observed between the samples in the Hep3B cell line. The Spearman values at the gene expression level had an average of 0.88, whereas at the transcript level it was on average of 0.70. The correlation matrix is presented in Supplementary File S1 (Figure S9).

**Figure 4:**
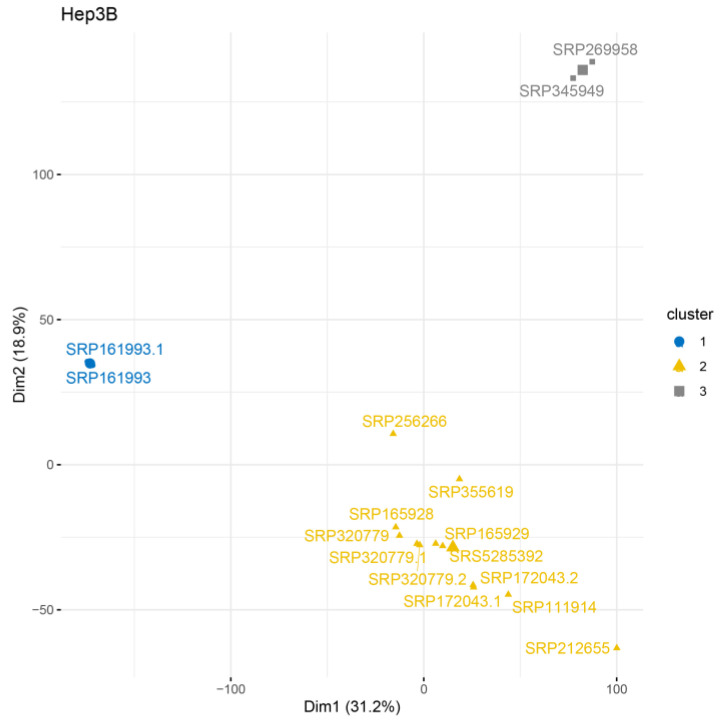
HCPC analysis at gene level for Hep3B cell line.

We identified three clusters of Hep3B cells based on their transcripts (Supplementary File S1, Figure S10) and gene levels (Figure 4). Notably, clusters #2 (yellow) and #3 (grey) contained samples from different studies, but cluster #1 (blue) included two samples from one study.

Furthermore, to gain deeper insight into the affected pathways across the three clusters, we conducted an analysis of differential gene expression. The results of the comparison for each pair of clusters are displayed in Table 4 and Supplementary Table S5, and Supplementary File S1 (Figure S11) shows the volcano and enrichment plots. The highest number of differentially expressed genes (3020) was observed between clusters #1 and #3.

A comparison of clusters #2 and #3 with cluster #1 revealed a disruption in the pathways “DNA IR-damage and cellular response via ATR” and “Retinoblastoma (RB) gene in cancer”, both involved in the regulation of the cell cycle and the DNA damage response, and their dysregulation can contribute to the development of cancer. The *CDK4* gene expression increases in cluster #2 compared to cluster #1 (log2fc = −2.44, FDR = 1.56 × $10^{-13}$) while *ATR* expression decreases (log2fc = 3.72, FDR = 1.10 × $10^{-7}$), which are included in these paths. The same genes are up- and down-regulated in cluster #3 compared to cluster #1: *CDK4* (log2fc = −3.04, FDR = 5.18 × $10^{-16}$) and *ATR* (log2fc = 4.86, FDR = 6.54 × $10^{-20}$).

Between clusters #2 and #3, there was a disruption in the DNA damage response pathways: “DNA damage response” (FDR = 0.01) and “Retinoblastoma (RB) gene in cancer pathway” (FDR = 0.001). The key genes of the latter pathway, *TP53* (log2fc = 3.02, FDR = 1.08 × $10^{-7}$) and *RB1* (log2fc = 4.61, FDR = 1.50 × $10^{-10}$) were increased in cluster #3 compared to cluster #2.

Between clusters #3 and #2, the pathways associated with folic acid and vitamin B12 metabolism were disrupted: “Folate metabolism” (FDR = 0.002) and “Vitamin B12 metabolism” (FDR < 0.001). The *TCN2* gene, involved in the vitamin B12 pathway, was downregulated in cluster #3 compared to cluster #2 (log2fc = −4.24, FDR = 5.52 × $10^{-12}$).

Hep3B cells originate from liver tissue, which is the main site for the synthesis of blood clotting factors. Between clusters #3 and #2, the “Blood clotting cascade pathway” changed (FDR < 0.001). The Hep3B cell line was used to evaluate the effects of coagulation factors on autophagy [14].

Changes in the “Network map of the SARS-CoV-2 signaling pathway” (FDR = 0.001) between the clusters #3 and #2 can be explained by changes in the expression of genes associated with the metabolism of folic acid, vitamin B12, or selenium in these cells since SARS-CoV-2 may influence the activities of these pathways. Therefore, folic acid may be an antagonist of SARS-CoV-2; however, its effect on viruses remains unclear [15,16].

### 2.2. Comparison of Liver Cell Lines 

As a result of transcriptomic profiling of HepG2 cell line, on average, 14,609 protein-coding genes and 37,844 transcripts (TPM > 0.1) were expressed. The Huh7 cell line has 14,879 genes and 38,662 transcripts, while Hep3B has 13,940 genes and 35,864 transcripts (TPM > 0.1). We performed a correlation analysis to study the similarity between the liver cell lines. Spearman correlation values were high for both gene and transcript expression. At the gene level, the average levels of Spearman correlation for Huh7 and HepG2 samples were 0.95, while when comparing transcript expression, it was 0.92 (Supplementary File S1, Figure S12). Similar values were observed when comparing Huh7 and Hep3B cells (gene correlation = 0.95, transcript correlation = 0.93) and HepG2 and Hep3B cells (gene correlation = 0.95, transcript correlation = 0.92).

Liver cell lines were also compared with cell lines that originated from other tissue types: A549 (lung cancer), HeLa (cervical cancer), and HEK293T (kidney cancer) as outgroups. The transcriptomic data for these cell lines were also searched in the NCBI SRA database (https://www.ncbi.nlm.nih.gov/sra, accessed on 5 September 2022) by criteria: total first/second fragment count >20,000,000; average read length >60 base pairs (bp); non-treated cells; Illumina sequencing technology. The accession numbers for the experiments are provided in Supplementary Table S2. The average correlation for the HepG2/Huh7/Hep3B and A549/HeLa/HEK293T pairs at the gene expression level was 0.82 and at the transcriptome level it was 0.78 (Supplementary File S1, Figure S12).

**Figure 5:**
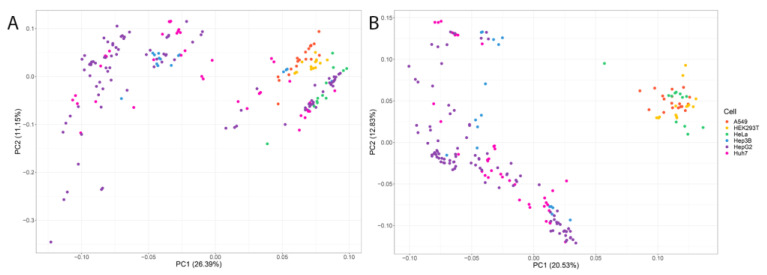
PCA analysis for six cell lines (**A**) at gene expression level; (**B**) at transcript expression level.

The next step was to run a PCA to determine how the cell lines clustered according to expression levels. We found that liver cell lines did not form distinct clusters, both at the level of gene expression and at the level of transcripts (Figure 5A,B). The use of such data for studying differential gene expression can lead to false results; therefore, we omitted this stage of cell line analysis.

In addition, samples of A549, HEK293T, and HeLa cell lines were included in the PCA. As a result of PCA at the level of transcript expression, we observed that the hepatic lines clustered into one cluster and the rest of the lines into another cluster. The cell lines divide randomly at the gene expression level.

This could be explained by the fact that all cells share basic biological processes, such as DNA replication and transcription, that are regulated by similar sets of genes. However, when we descended to the transcriptome level, the differences in transcriptome expression increased.

### 2.3. Differences between Hepatic Cell Lines and Primary Hepatocytes

Primary hepatocytes (PHP) are liver cells that are isolated directly from tissue. In contrast, liver cell lines such as HepG2, Huh7, and Hep3B are derived from hepatoblastoma and hepatocellular carcinoma cells, respectively. Thus, comparison of hepatic cell lines with primary hepatocytes can provide insight into similarities and differences in gene expression and pathway activation between these cell types.

The transcriptomic data for primary hepatocytes was searched in the NCBI SRA database (https://www.ncbi.nlm.nih.gov/sra, accessed on 3 May 2023) according to the criteria specified above. For the primary hepatocytes, 31 samples were found. The accession numbers for the experiments are provided in Supplementary Table S2. Searches for hepatocyte-specific datasets yielded limited results due to the inclusion of studies that used liver cell lines rather than primary hepatocytes or that used hepatocytes from diseased donors. The average total number of reads and average length were 64,553,776 and 129 bp, respectively. As a result of transcriptome profiling of primary hepatocytes, on average, 15,173 protein-coding genes and 39,018 transcripts were expressed with a TPM greater than 0.1.

**Figure 6:**
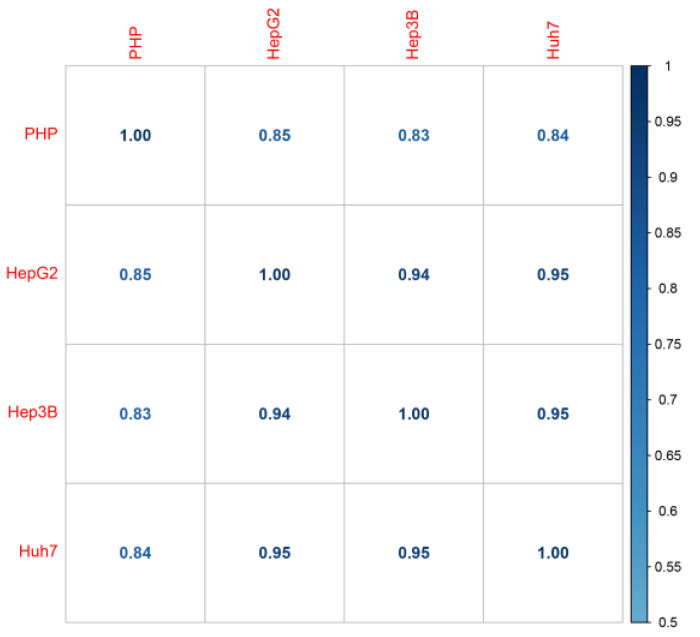
Spearman correlation of gene expression: hepatic cell lines and primary hepatocytes (PHP).

We compared liver cell lines and primary hepatocytes in terms of gene and transcript expression. The Spearman correlation between the average expression levels of all HepG2 and PHP samples at the gene level was 0.85 (Figure 6), while it was 0.82 when comparing transcript expression (Supplementary File S1, Figure S13). Similar results were obtained when comparing Huh7 and PHP cells (gene expression = 0.83, transcript expression = 0.82) and Hep3B and PHP (gene expression = 0.84, transcript expression = 0.81).

**Figure 7:**
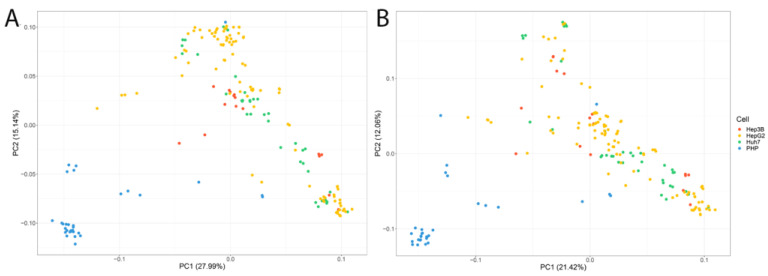
PCA analysis for three liver cell lines and primary hepatocytes (PHP) (**A**) at gene expression level; (**B**) at transcript expression level.

The next step was to run a PCA to determine how the cell lines clustered according to gene and transcript expression levels. As a result of the analysis, we found that primary hepatocytes did not mix with hepatic cell lines but formed their own separate clusters both at the level of gene and transcript expression (Figure 7A,B).

As a result of comparing hepatic cell lines with primary hepatocytes, we found that the largest number of differentially expressed genes were detected when comparing Hep3B with PHP. The results of the comparison are displayed in Table 5 and Supplementary Table S6, and Supplementary File S1 (Figure S14) shows the volcano and enrichment plots.

The comparative transcriptomic analysis of HepG2, Hep3B, and Huh7 with primary hepatocytes revealed significant differences in pathways related to cell cycle, such as “G1 to S cell cycle control” and “Cell cycle”. Disruptions in these pathways lead to uncontrolled cell proliferation. The cell cycle is regulated by cyclin-dependent kinases [17], which are upregulated in tumor cells. Our analysis observed upregulation of *CDK2* and *CDK4* genes in hepatic cell lines compared to primary hepatocytes.

The “Retinoblastoma gene in cancer” pathway is crucial in regulating the cell cycle and preventing uncontrolled cell growth. Our analysis showed that this pathway is perturbed in the three cell lines (HepG2, Hep3B, and Huh7) compared to primary hepatocytes (PHP). Dysregulation of the RB pathway is strongly associated with the development of cancer. Notably, key genes involved in this pathway, such as *E2F1* and *CKS2*, are upregulated in the cell lines compared to PHP. E2F1 is a transcription factor that plays a vital role in cell cycle regulation and cell proliferation. Its dysregulation can lead to uncontrolled cell growth and promote cancer. Similarly, CKS2 regulates the cell cycle by controlling the activity of cyclin-dependent kinases. Overexpression of *CKS2* is observed in many types of cancer and is thought to promote the proliferation and survival of cancer cells. Conversely, the tumor suppressor gene *PTEN*, also involved in this pathway, is suppressed in all cell lines compared to PHP.

Additionally, in all cell lines, the “PPAR signaling” pathway, which is involved in the regulation of lipid metabolism and glucose homeostasis, is affected. This pathway was found to play a crucial role in liver diseases, including non-alcoholic fatty liver disease, cirrhosis, and hepatocellular carcinoma [18]. One of the key genes of this pathway, *PPARA*, is downregulated in all liver cell lines compared to PHP [19]. PPARs are nuclear receptors that regulate lipid and glucose metabolism in the liver. Dysregulation of PPAR signaling can lead to lipid accumulation, inflammation, and oxidative stress, which are associated with liver disease development.

**Figure 8:**
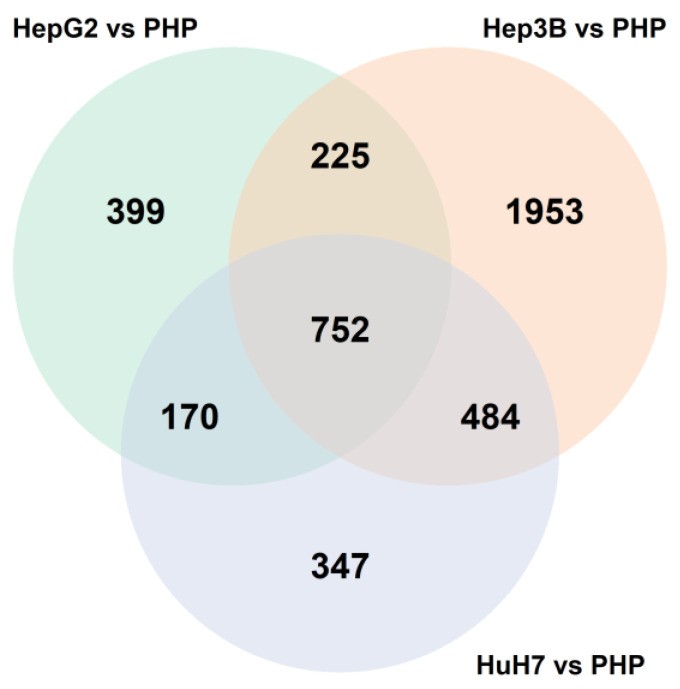
Comparison of significant differentially expressed genes for three hepatic cell lines and primary hepatocytes (PHP).

Furthermore, to assess the differences between three liver cell lines and primary hepatocytes (PHP), we identified 752 common genes by combining all statistically significant differentially expressed genes between hepatic cell lines and PHP (Figure 8). Of note, we observed that Hep3B and Huh7 cells have a greater similarity in their molecular profiles in comparison with hepatocytes, while Huh7 and HepG2 cells have the least similarity.

An enrichment analysis of differentially expressed genes was conducted. To perform the enrichment analysis, we used the PANTHER database (http://pantherdb.org/, accessed on 5 May 2023). Our analysis revealed that the genes were involved in several biological processes, including “Cell cycle” (FDR = 1.29 × $10^{-16}$), “Mitotic cell cycle” (FDR = 1.50 × $10^{-16}$), and “Regulation of the cell cycle process” (FDR = 5.45 × $10^{-13}$). Moreover, these genes participated in “DNA replication” (FDR = 4.22 × $10^{-17}$), “DNA repair” (FDR = 5.49 × $10^{-21}$), and “DNA metabolic processes” (FDR = 1.44 × $10^{-11}$). We also found that these genes were involved in the metabolism of carbohydrates, lipids, and amino acids—“Small molecule metabolic processes” (FDR = 4.49 × $10^{-19}$).

We report that the *HSD17B6* and *HSD17B13* genes are suppressed in liver cell lines compared to primary hepatocytes. Reduced *HSD17B6* expression correlates with tumor stage and grade in hepatocellular carcinoma [20]. *HSD17B13*, on the other hand, is involved in steroid hormone metabolism, and mutations in this gene are associated with non-alcoholic fatty liver disease and insulin resistance. Of note, *HSD17B13* knockout in HepG2 cells does not affect their lipid content, suggesting that this gene does not directly regulate lipid content in this cell line [21].

Another important gene associated with liver function is *ABCB11*, which encodes a protein involved in the transport of bile acids from the liver to the small intestine. This gene plays a critical role in maintaining proper liver function.

Most of the genes that are upregulated in liver cell lines play a crucial role in various cellular processes such as cell migration, adhesion, and differentiation. For example, *SEMA6B* is a gene that is expressed in the liver and encodes a protein involved in cell migration. Upregulation of *SEMA6B* in cancer may promote tumor growth and metastasis by increasing cell migration and invasion [22]. *GDF15* is involved in the regulation of cell proliferation and differentiation, while *MDK* is a multifunctional cytokine that promotes angiogenesis and regulates cell migration.

## 3. Discussion

Between clusters of the HepG2 cell line, we observed changes in the pathways that are responsible for the synthesis of proteins involved in the metabolism of drugs and other xenobiotics. The HepG2 cell line is often used as a model system for drug metabolism analyses. We observed reduced expression of genes associated with pathways involved in drug metabolism and detoxification, such as *CYP3A5*, *CYP20A1*, *SULT1A4*, and *SULT1A2*. The reduced expression of these genes indicates that cells may have a reduced capacity for drug metabolism and detoxification, potentially affecting the accuracy and relevance of drug metabolism analyses performed using HepG2 cells.

Understanding these differences is important for researchers using HepG2, Huh7, and Hep3B cell lines as model systems to study various biological processes, including drug metabolism, gene regulation, and liver disease. It also shows the need to standardize protocols for growing cell lines and the reagents used.

The comparison of liver cell lines revealed that they formed a single cluster based on gene expression and transcripts. However, it is important to note that the HepG2, Huh7, and Hep3B lines correspond to different cancer subtypes, and their overall molecular profiles may not differ significantly. Nevertheless, each of these cell lines may have distinct driver genes. Therefore, a comprehensive analysis of these cell lines, including mutation profile, proteomics, metabolomics, and histological features, is necessary.

When comparing hepatic lines with non-hepatic ones, we did not observe a difference in the gene expression level. However, the lines were divided into two clusters at the transcript level: non-hepatic cell lines were grouped into one cluster and hepatic cells into another. Moreover, it can be assumed that the differences at the proteomic and metabolic levels will be more pronounced, especially given the differences observed between the passages of the HepG2 cell line [23].

The comparative transcriptomic analysis of liver cell lines and primary hepatocytes revealed significant differences in key pathways related to cell cycle control, such as “G1 to S cell cycle control” and “Cell cycle” pathways. These pathways in normal cells regulate cell growth, prevent uncontrolled cell proliferation, and maintain liver function. We also observed dysregulation in the PPAR signaling pathway, which is linked to non-alcoholic fatty liver disease, cirrhosis, and hepatocellular carcinoma. *PPARA*, one of the key genes involved in this pathway, is downregulated in all cell lines compared to primary hepatocytes.

In conclusion, our study revealed significant differences in gene expression between hepatic cell lines and primary hepatocytes. We identified 747 common differentially expressed genes in three popular hepatic cell lines compared to PHP associated with cell cycle processes. Taken together, this gives insight into the potential limitations of using hepatic cell lines as a model for primary hepatocytes and emphasizes the importance of careful choice of the model system in liver-related studies.

## 4. Methods and Materials

### 4.1. Sequencing of IBMC Data 

The cells were obtained from Sigma–Aldrich and grown in DMEM/F12 supplemented with 10% fetal bovine serum and penicillin/streptomycin. The total RNA was isolated from the HepG2 cell line using a RNeasy mini kit, and the quality of the RNA was assessed using a Bioanalyzer 2100 System. The mRNA extraction was conducted with a Dynabeads™ mRNA purification kit, and the mRNA was quantified using a Qubit 4 fluorometer and a Qubit RNA HS assay kit. The sequencing was performed using the Illumina NovaSeq 6000 with a read length of 100 bp, and the TruSeq Stranded mRNA library prep kit was used to prepare RNA libraries. The transcriptomic profiling was performed in three replicates with a time-division RNA process. The full protocol can be found in Pyatnitskiy et al. [6].

### 4.2. Search of Public Data 

The search of public RNAseq data was performed in the NCBI SRA database (https://www.ncbi.nlm.nih.gov/sra, accessed on 5 September 2022) by criteria: total first/second fragment count >20,000,000; average read length >60 base pairs (bp); non-treated cells; Illumina sequencing technology. Accession numbers for the experiments found are provided in Supplementary Table S2.

### 4.3. Data Analysis

Quality control was performed using FastQC [24]. Trimming of reads was carried out using Trim Galore (version 0.6.7) with the following arguments: -q 20 -length 36. Gene expression and isoform analysis were performed using Salmon (mapping-based mode, version 1.6.0) [25]. The transcript expression was quantified in transcript per million (TPM) units. The gene expression was calculated by summing all the TPMs of the corresponding transcripts.

As a genomic annotation, we used the GTF file downloaded from the Ensembl resource (GRCh38, release 103). We limited our analysis to protein-coding genes and did not include transcripts assigned to the non-standard chromosomes, resulting in 60,740 protein-coding transcripts and 19,670 genes.

The R software environment was used for computations and visualization (ver. 4.1) [26]. We used several packages for the analysis including the FactoMineR [27] for hierarchical clustering on principal components (HCPC), the DESeq2 [5] for differential expression, the EnhancedVolcano for volcano plots (https://github.com/kevinblighe/EnhancedVolcano, accessed on 1 February 2023), and ClusterProfiler [28] for gene set enrichment.

Upon performing PCA analysis, we observed that samples from one study were separated into distinct clusters. To address this issue, we utilized the Combat method to correct for the batch effect [29]. However, despite applying this correction, we did not observe any significant changes in the results, indicating that the batch effect was not the primary cause of the observed clustering.

## 5. Conclusions

Our study aimed to analyze the heterogeneity among liver cell lines—HepG2, Huh7, and Hep3B. In brief, we found that HepG2, Huh7, and Hep3B cell lines differed in oxidative phosphorylation, cholesterol metabolism, and DNA damage between samples and cell lines. In addition, despite the high correlation of gene expression between samples of the same cells lines, a batch effect was noted, which is likely due to the cultivation conditions. Thus, in order to transfer the results of experiments to other cell line samples, many factors must be considered, including growing conditions such as nutrient and oxygen concentrations, pH of the medium, and other parameters, which may lead to inaccurate or distorted data.

A notable fact is that when comparing cell lines of different origins based on gene expression, the difference between them is not obvious, while the use of transcript expression gives a clear separation by cell source. Together with our previous results on the difference in the metabolomic profile between the passages of HepG2 cells, it indicates that the closer the omics layer is to the phenotype, the greater the differences between the cells will be observed.

Comparative transcriptomic analysis of liver cell lines and primary hepatocytes shows that these cells have a common difference in their cell cycle processes. Interestingly, there are the most differentially expressed genes in the Hep3B cell line compared to primary hepatocytes. Thus, using liver cell lines as a model for normal hepatocytes may not be appropriate in some experiments, as key pathways involved in cancer development and liver disease are affected in HepG2, Huh7, and Hep3B cell lines.

## Figures and Tables

**Table 1:** Available transcriptome data matching specified criteria.

Cell Lines	Number of Studies	Number of Samples	Average Total Number of Reads	Average Read Length (bp)
HepG2	47	97	30,676,937	130
Huh7	21	39	30,561,171	126
Hep3B	12	16	34,473,198	129
HepG2 IBMC data	2	2	69,637,946	100

**Table 2:** Number of genes with differential expression between the clusters of HepG2 cell lines.

Clusters	Sum of Differential Expression Genes	Up	Down
#2 vs. #1	1093	196	897
#3 vs. #1	2871	1179	1692
#2 vs. #3	2085	9532	1133

**Table 3:** Number of genes with differential expression between the clusters of Huh7 cell lines.

Clusters	Sum of Differential Expression Genes	Up	Down
#2 vs. #1	1032	854	178
#3 vs. #1	2923	1703	1220
#4 vs. #1	3575	2132	1443
#3 vs. #2	2130	1056	1074
#4 vs. #2	1771	958	813
#3 vs. #4	816	440	376

**Table 4:** Number of genes with differential expression between the clusters of Hep3B cell lines.

Clusters	Sum of Differential Expression Genes	Up	Down
#2 vs. #1	2904	1068	1836
#3 vs. #1	3020	1265	1755
#3 vs. #2	404	118	286

**Table 5:** Number of genes with differential expression between the three hepatic cell lines and primary hepatocytes (PHP).

	Sum of Differential Expression Genes	Up	Down
HepG2 vs. PHP	1562	715	847
Huh7 vs. PHP	1767	625	1142
Hep3B vs. PHP	3442	1961	1481

## Data Availability

Raw transcriptome data files are publicly released on the NCBI SRA (https://www.ncbi.nlm.nih.gov, accessed on 17 April 2023). The accession number is PRJNA765908.

## Supplementary Materials

The following supporting information can be downloaded at: https://www.mdpi.com/article/10.3390/ijms24108791/s1.
