# Supplementary material for: Comparative Transcriptomic Analysis of Three Common Liver Cell Lines
Source: Int J Mol Sci. 2023 May 15;24(10):8791. doi: 10.3390/ijms24108791 (PMC10218629; doi:10.3390/ijms24108791)
Supplement: Supplementary file 1 [file ijms-24-08791-s001.zip › ijms-2379862-supplementary/Supplemetary File S1.pdf]

Table S1. IBMC data quality for each sample.

Sample	Repeats	Data	Num Total Reads	Average Read Length	Accession Number
IBMC 1	IBMC 1.1	2020	59,789,684	100	SRR16071314
	IBMC 1.2		95,048,978	100	SRR16071312
	IBMC 1.3		54,075,176	100	SRR16071311
IBMC 2	IBMC 2.1	2021	63 796 026	99	SRR24201566
	IBMC 2.2		77 281 718	99	SRR24201565
	IBMC 2.3		71 212 496	99	SRR24201564

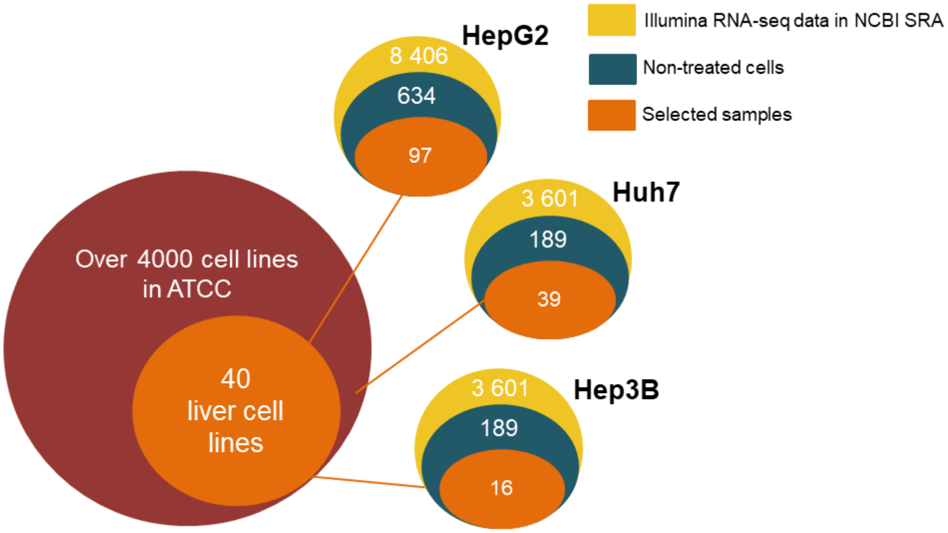

Figure S1. Available RNAseq data for HepG2, Hep3B и Huh7 selected for the study.

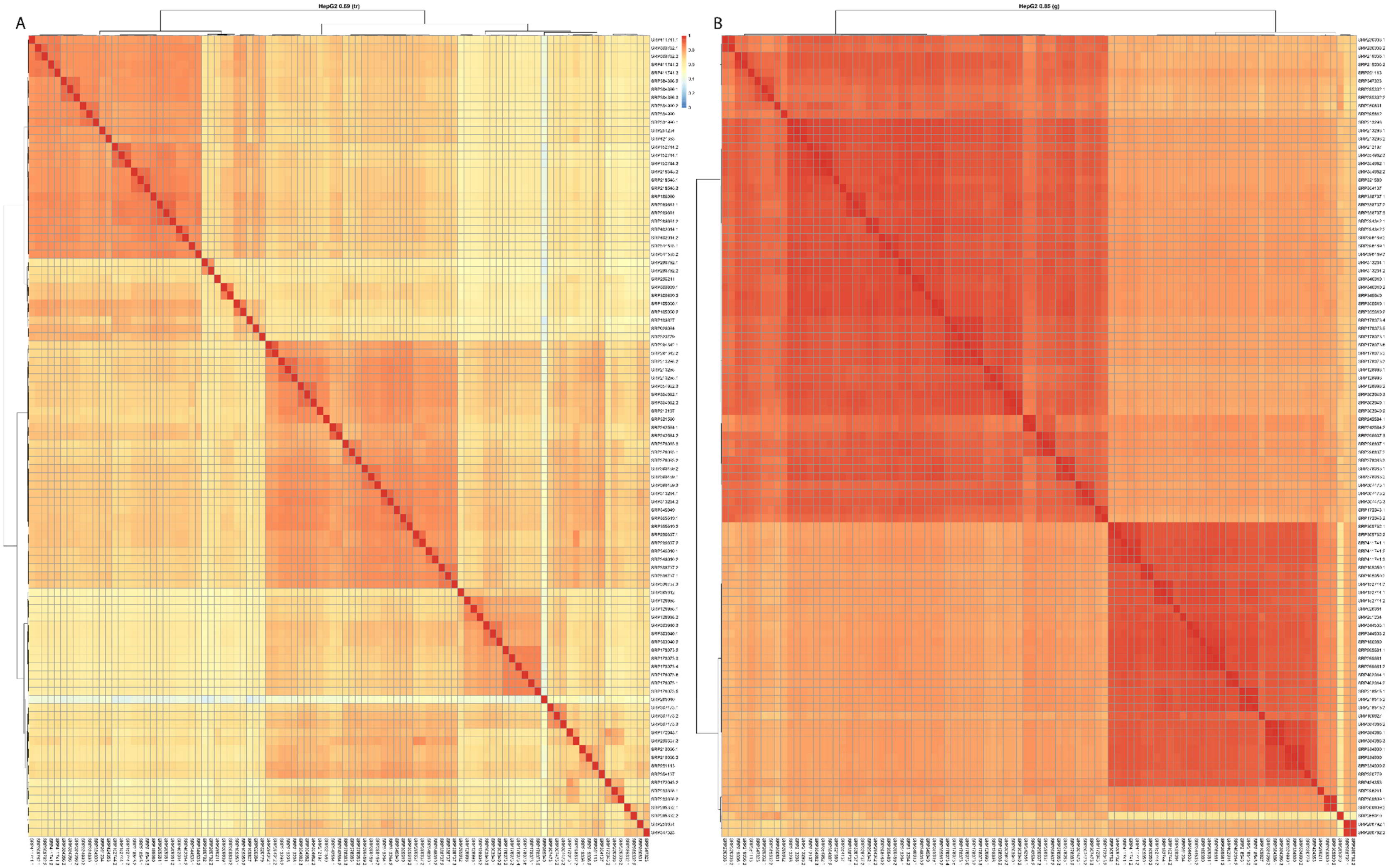

Figure S2. (A) Correlation matrix between transcripts; (B) Correlation matrix between genes.

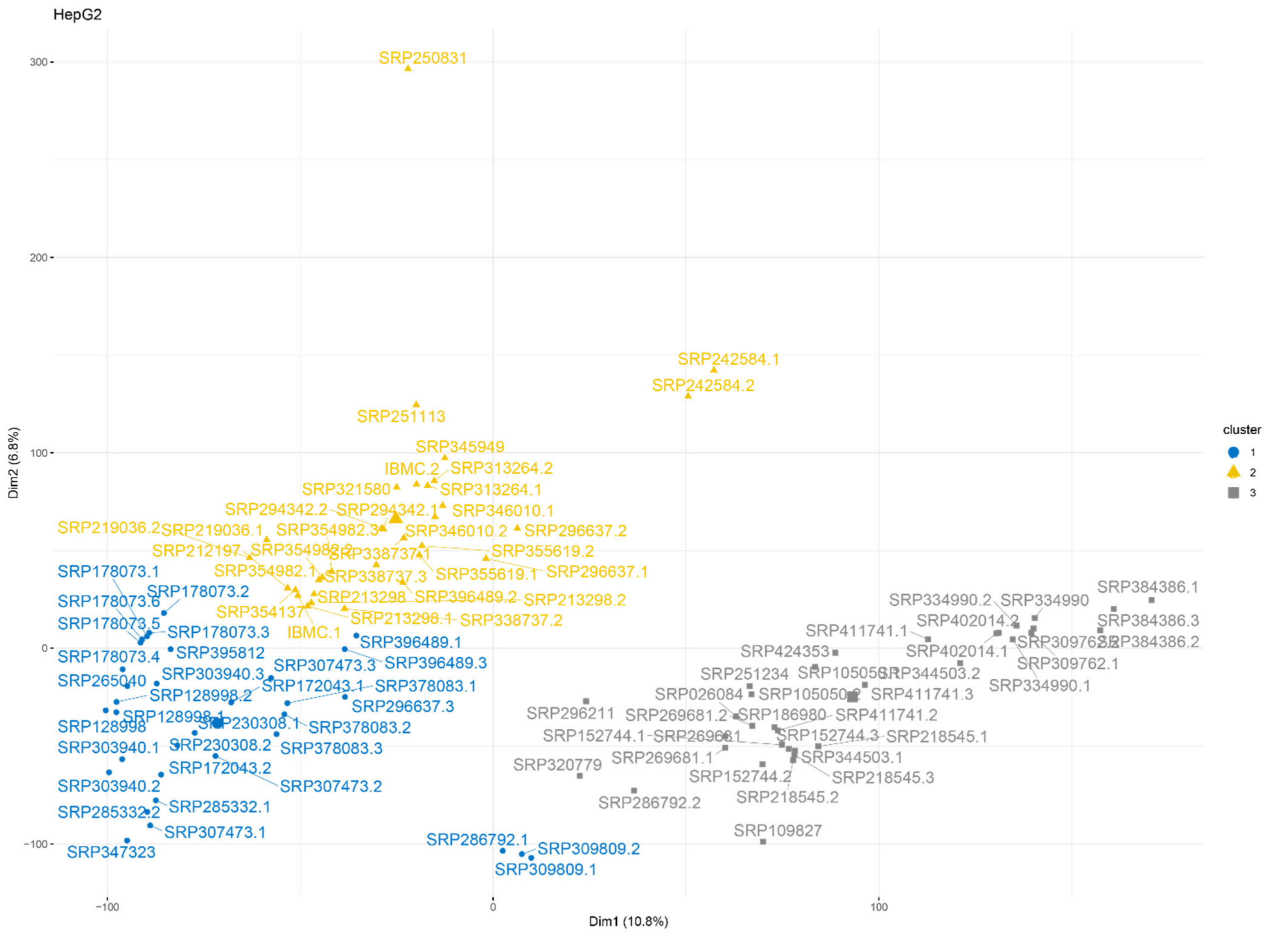

Figure S3. HCPC analysis on transcripts level for HepG2 cell line.

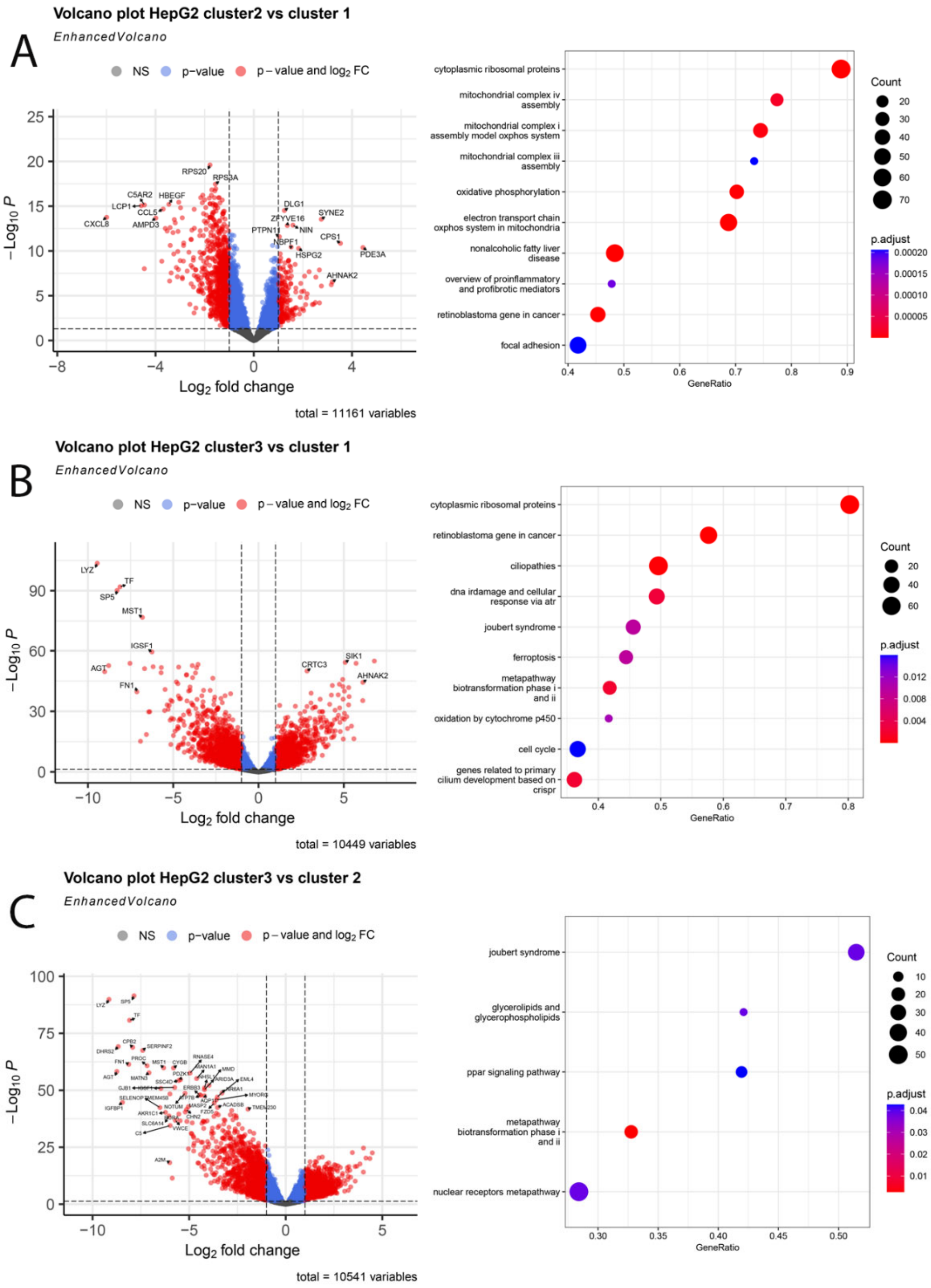

Figure S4. Transcriptomic profiling of HepG2 cell line. (A) Volcano plot showing differentially expressed genes between HepG2 clusters two and one (left figure). Gene Set Enrichment Analysis of differentially expressed genes between HepG2 clusters two and one (right figure). Core genes is the subset of genes in the geneset contributing to the enrichment signal.

(B) Volcano plot showing differentially expressed genes between HepG2 clusters three and one (left figure). Gene Set Enrichment Analysis of differentially expressed genes between HepG2 clusters three and one (right figure). Core genes is the subset of genes in the geneset contributing to the enrichment signal.

(C) Volcano plot showing differentially expressed genes between HepG2 clusters three and two (left figure). Gene Set Enrichment Analysis of differentially expressed genes between HepG2 clusters three and two (right figure). Core genes is the subset of genes in the geneset contributing to the enrichment signal.

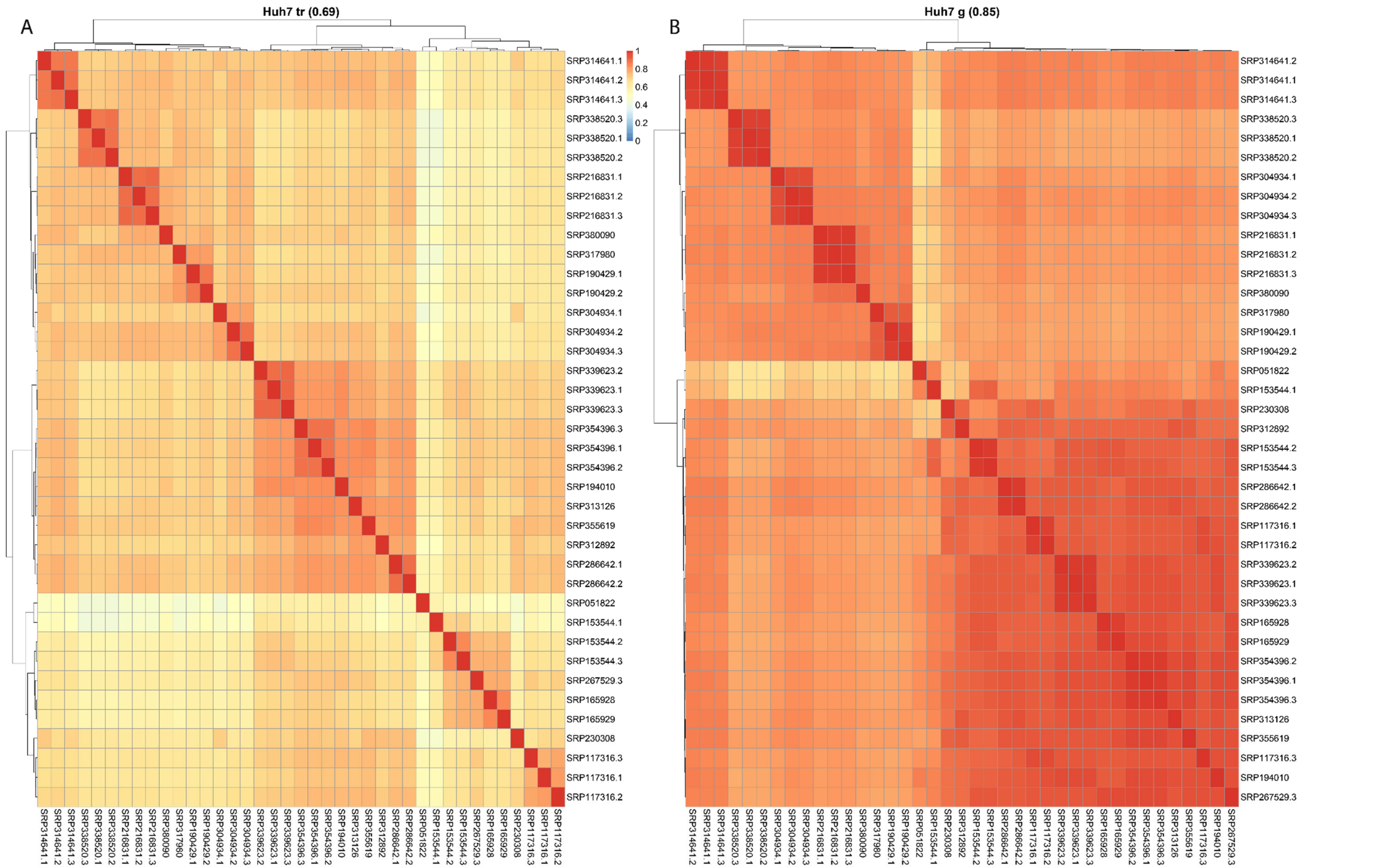

Figure S5. (A) Correlation matrix between transcripts; (B) Correlation matrix between genes.

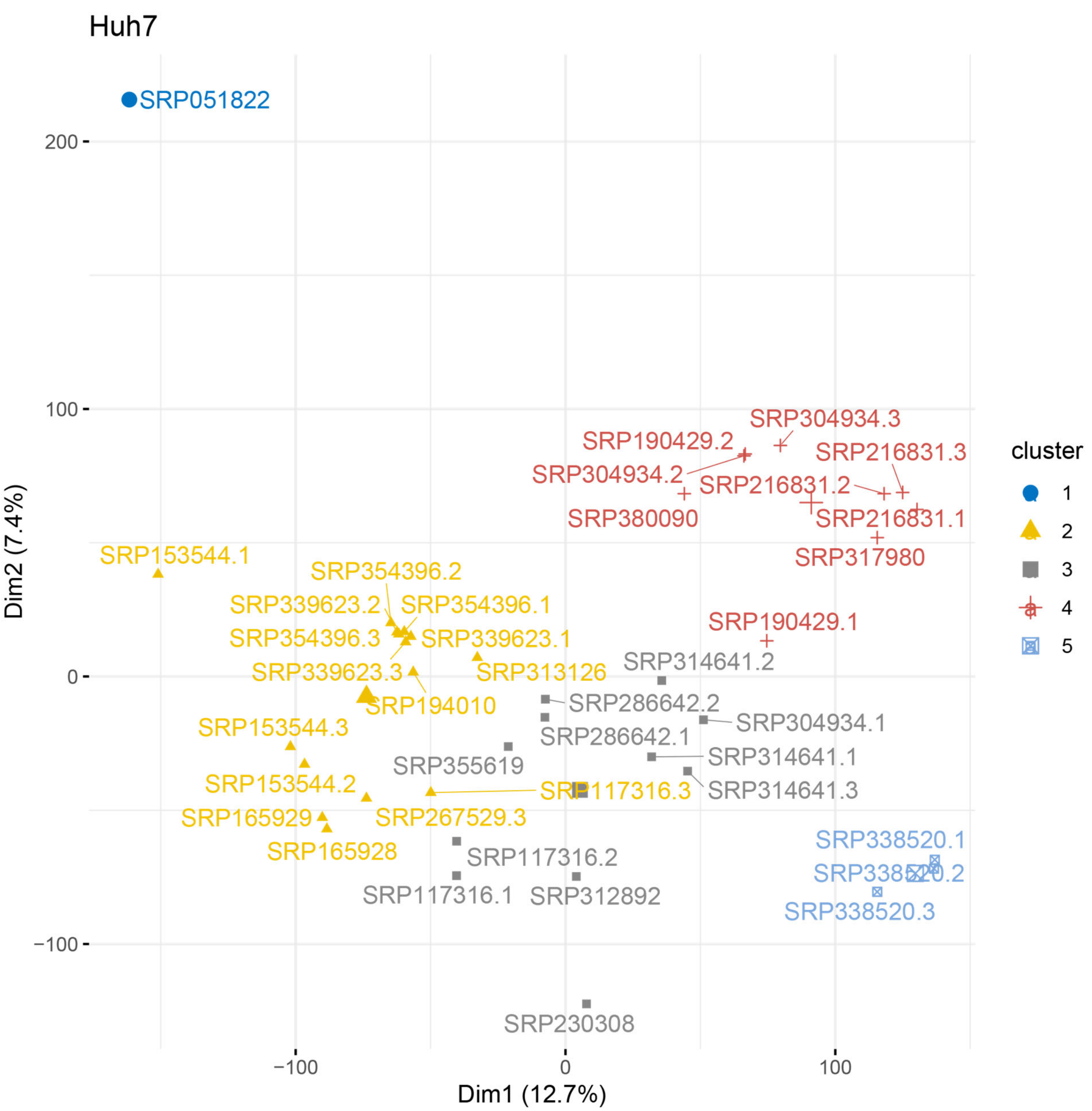

Figure S6. HCPC analysis on transcripts level for Huh7 cell line.

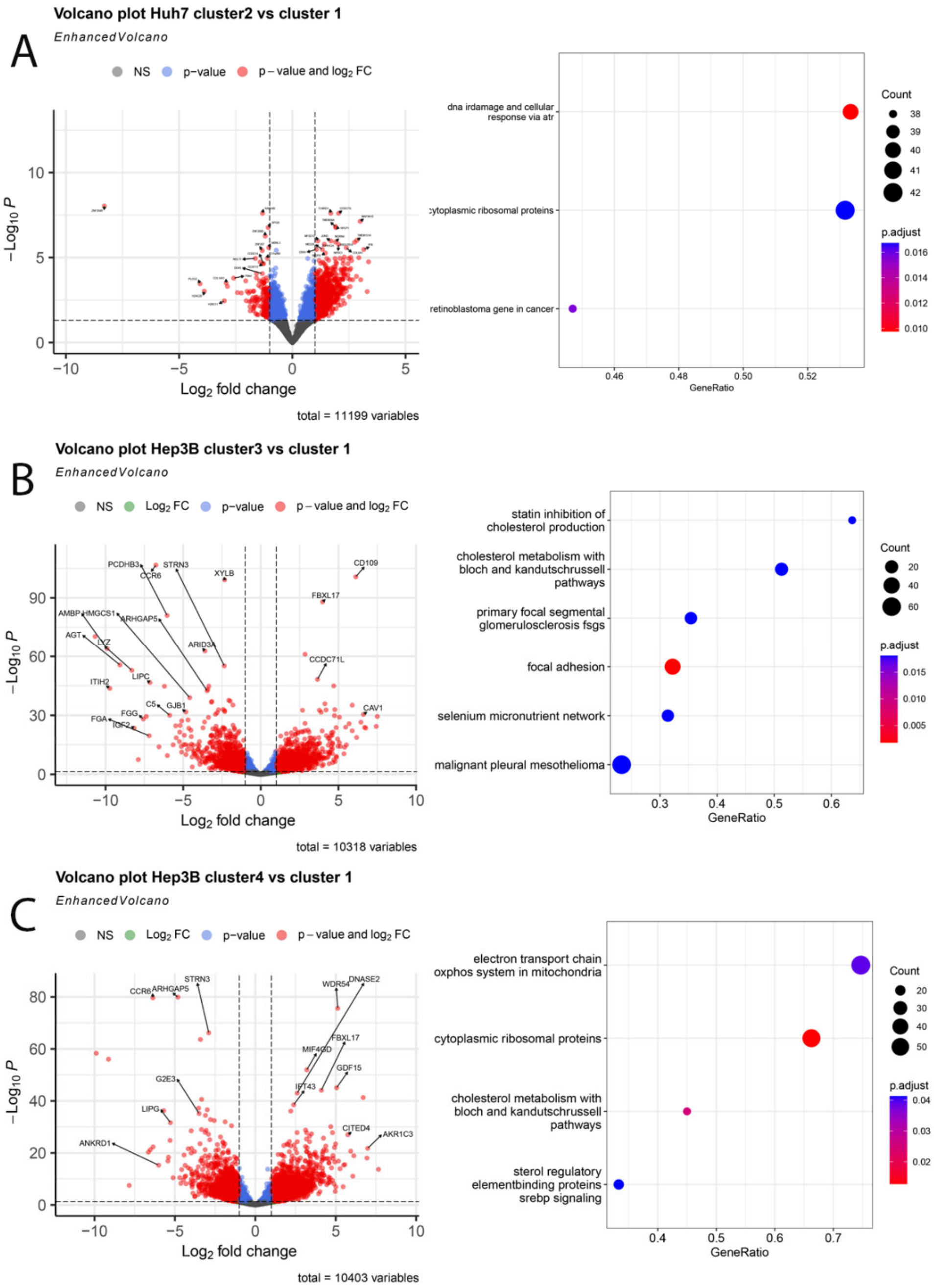

Figure S7. Transcriptomic profiling of Huh7 cell line. (A) Volcano plot showing differentially expressed genes between Huh7 clusters two and one (left figure). Gene Set Enrichment Analysis of differentially expressed genes between Huh7 clusters two and one (right figure). Core genes is the subset of genes in the geneset contributing to the enrichment signal.

(B) Volcano plot showing differentially expressed genes between Huh7 clusters three and one (left figure). Gene Set Enrichment Analysis of differentially expressed genes between Huh7 clusters three and one (right figure). Core genes is the subset of genes in the geneset contributing to the enrichment signal.

(C) Volcano plot showing differentially expressed genes between Huh7 clusters four and one (left figure). Gene Set Enrichment Analysis of differentially expressed genes between Huh7 clusters four and one (right figure). Core genes is the subset of genes in the geneset contributing to the enrichment signal.

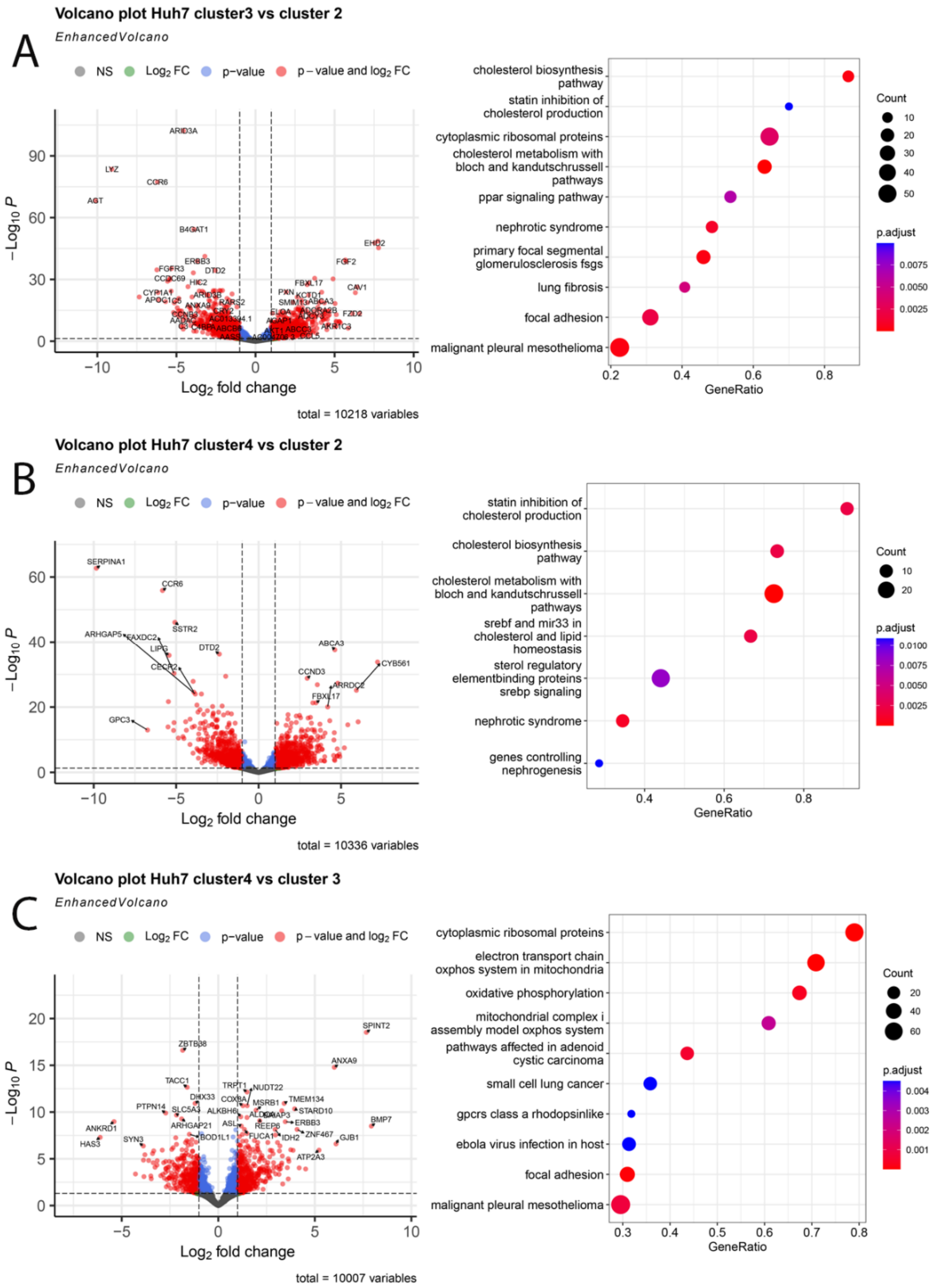

Figure S8. Transcriptomic profiling of Huh7 cell line. (A) Volcano plot showing differentially expressed genes between Huh7 clusters two and three (left figure). Gene Set Enrichment Analysis of differentially expressed genes between Huh7 clusters two and three (right figure). Core genes is the subset of genes in the geneset contributing to the enrichment signal.

(B) Volcano plot showing differentially expressed genes between Huh7 clusters two and four (left figure). Gene Set Enrichment Analysis of differentially expressed genes between Huh7 clusters two and four (right figure). Core genes is the subset of genes in the geneset contributing to the enrichment signal.

(C) Volcano plot showing differentially expressed genes between Huh7 clusters four and three (left figure). Gene Set Enrichment Analysis of differentially expressed genes between Huh7 clusters four and three (right figure). Core genes is the subset of genes in the geneset contributing to the enrichment signal.

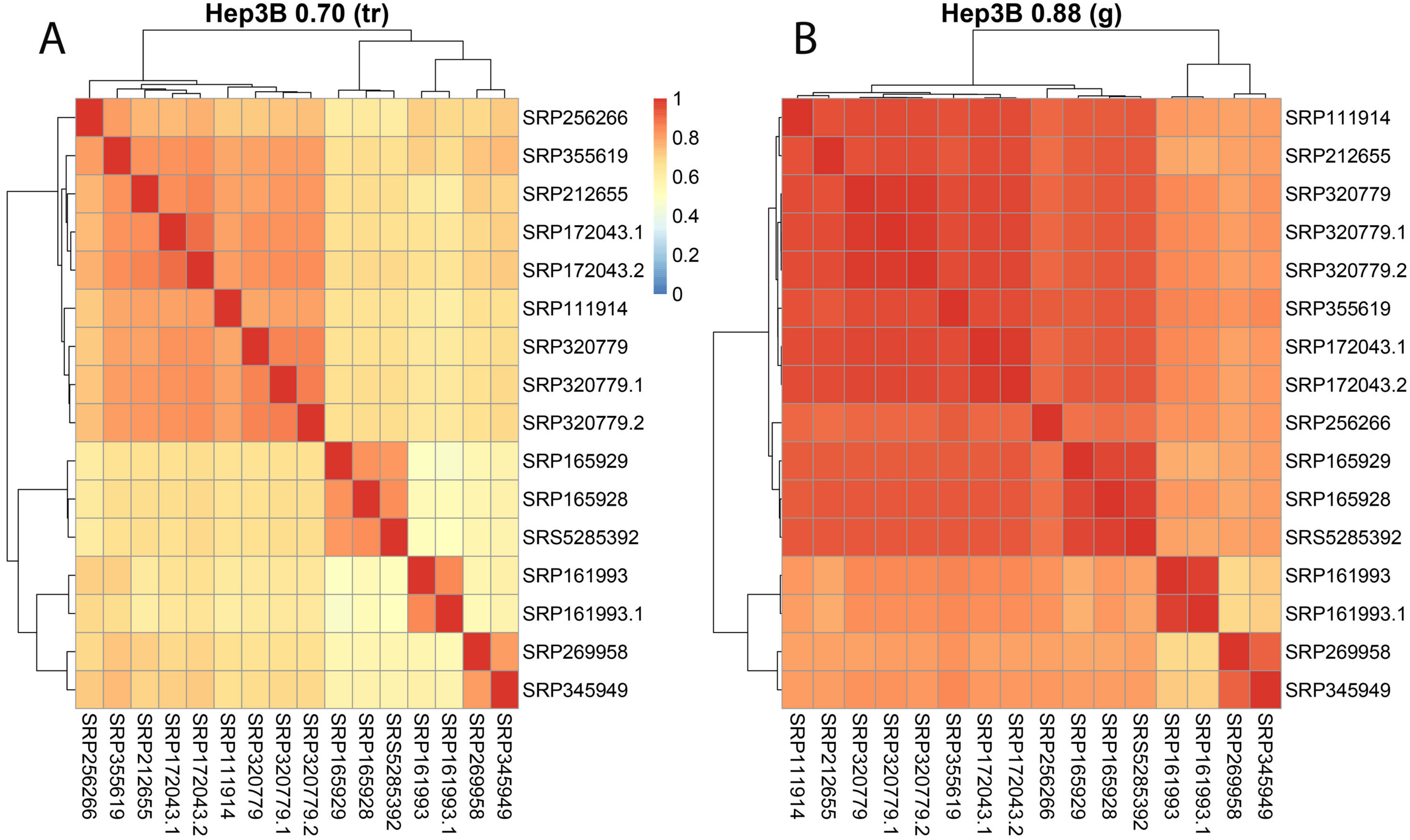

Figure S9. (A) Correlation matrix between transcripts; (B) Correlation matrix between genes.

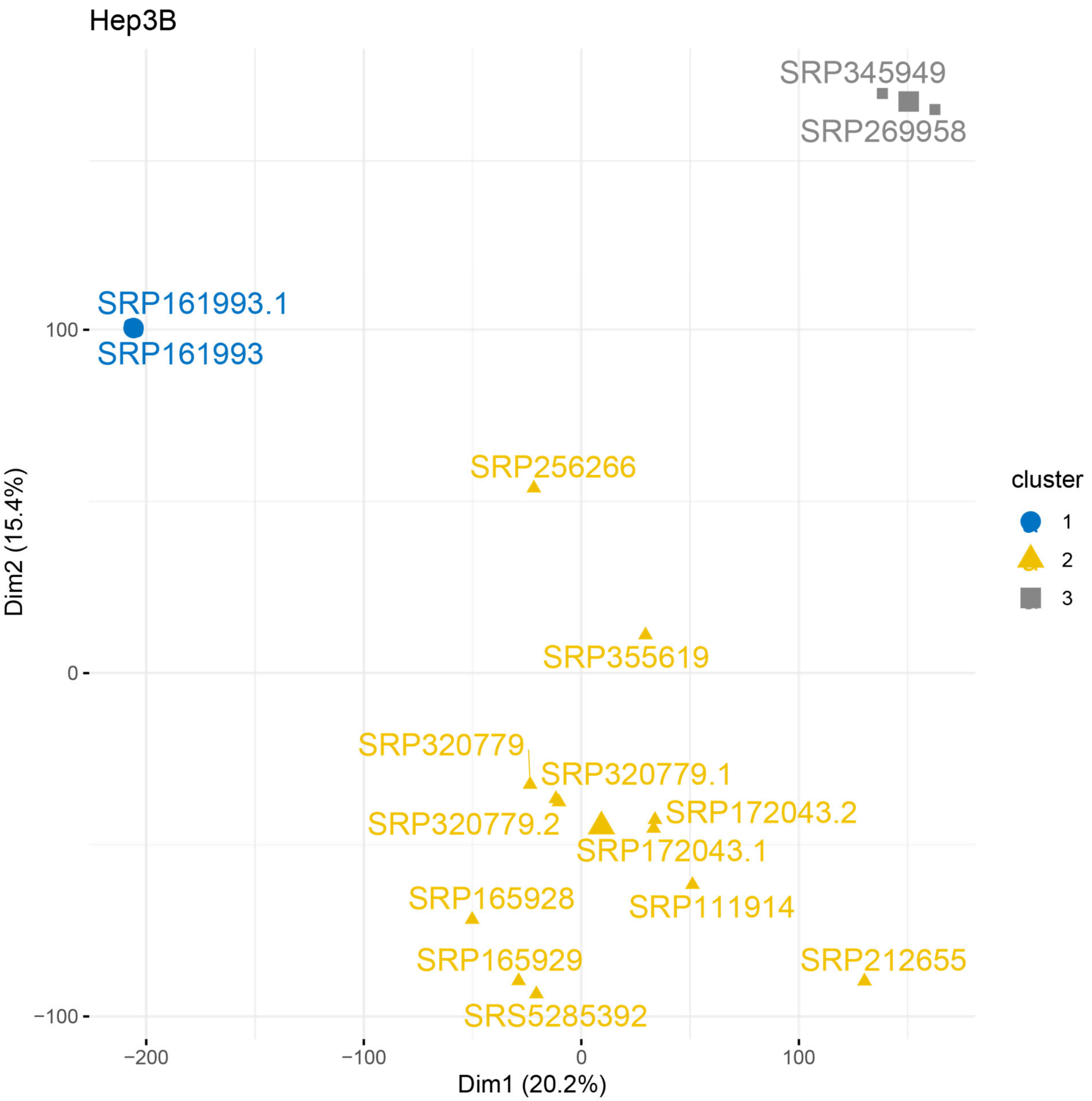

Figure S10. HCPC analysis on transcripts level for Hep3B cell line.

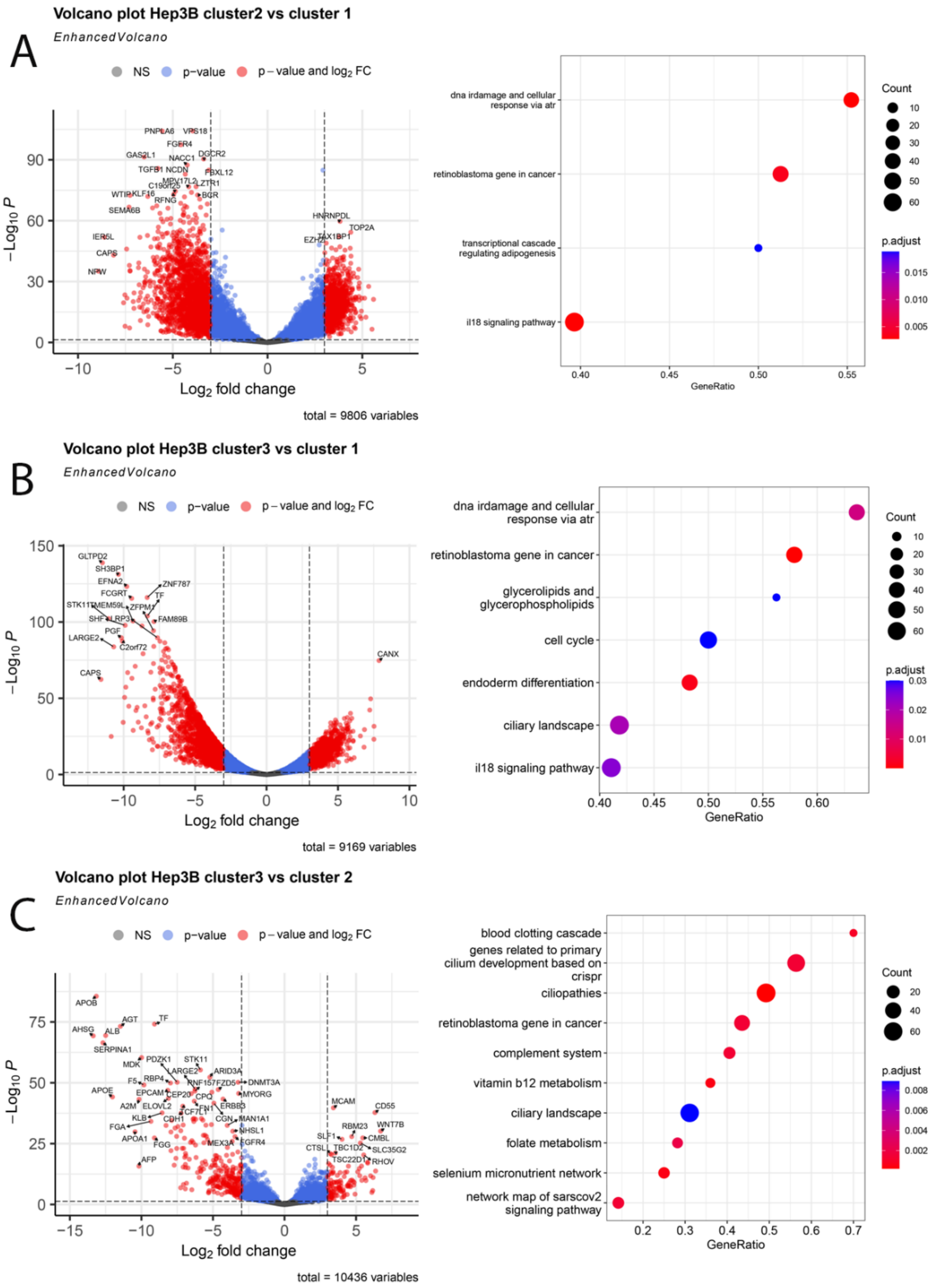

Figure S11. Transcriptomic profiling of Hep3B cell line. (A) Volcano plot showing differentially expressed genes between Hep3B clusters two and one (left figure). Gene Set Enrichment Analysis of differentially expressed genes between Hep3B clusters two and one (right figure). Core genes is the subset of genes in the geneset contributing to the enrichment signal.

(B) Volcano plot showing differentially expressed genes between Hep3B clusters three and one (left figure). Gene Set Enrichment Analysis of differentially expressed genes between Hep3B clusters three and one (right figure). Core genes is the subset of genes in the geneset contributing to the enrichment signal.

(C) Volcano plot showing differentially expressed genes between Hep3B clusters three and two (left figure). Gene Set Enrichment Analysis of differentially expressed genes between Hep3B clusters three and two (right figure). Core genes is the subset of genes in the geneset contributing to the enrichment signal.

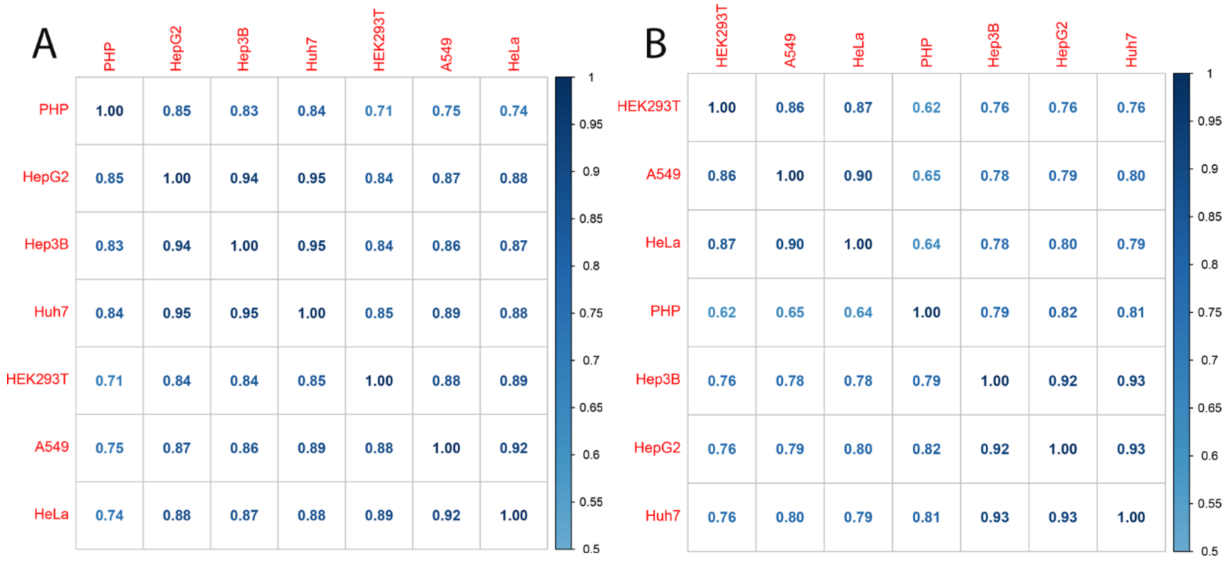

Figure S12. Expression of Spearman correlation table between three hepatic cell lines, primary hepatocytes (PHP) and A549 (lung cancer), HeLa (cervical cancer) and HEK293T (kidney cancer) A) genes expression level; B) transcripts expression level.

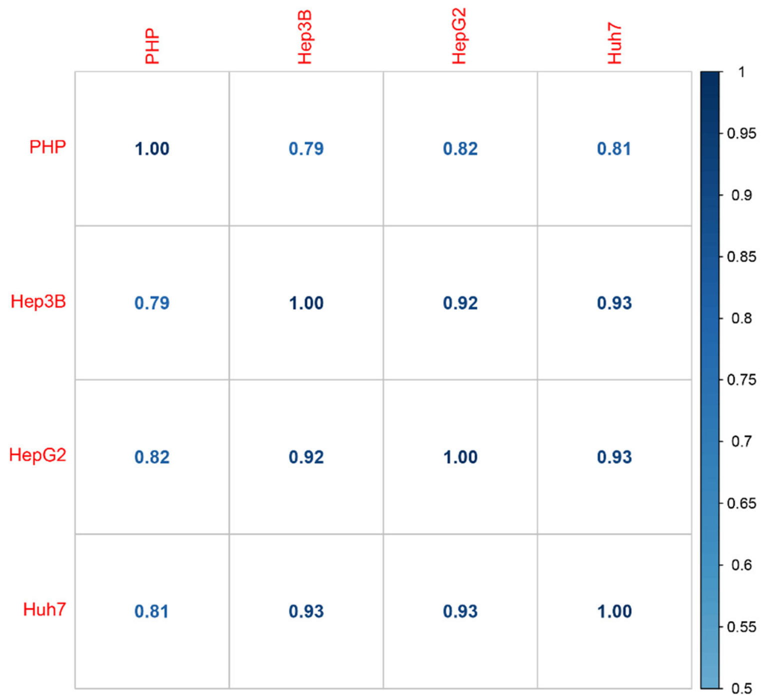

Figure S13. Transcripts expression of Spearman correlation table between hepatic cell lines and primary hepatocytes (PHP).

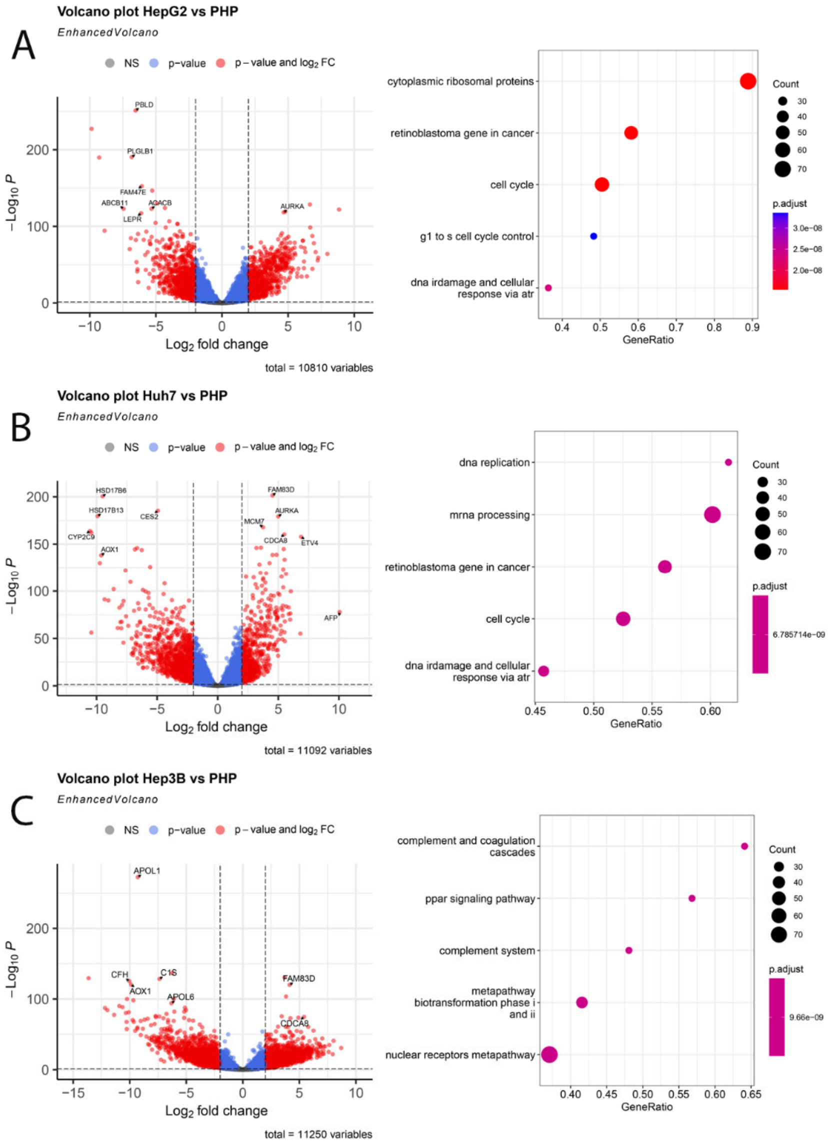

Figure S14. Transcriptomic profiling of primary hepatocytes (PHP) with three liver cell lines. (A) Volcano plot showing differentially expressed genes between HepG2 and PHP (left figure). Gene Set Enrichment Analysis of differentially expressed genes between HepG2 and PHP (right figure). Core genes is the subset of genes in the geneset contributing to the enrichment signal.
(B) Volcano plot showing differentially expressed genes between Huh7 and PHP (left figure). Gene Set Enrichment Analysis of differentially expressed genes between Huh7 and PHP (right figure). Core genes is the subset of genes in the geneset contributing to the enrichment signal.
(C) Volcano plot showing differentially expressed genes between Hep3B and PHP (left figure). Gene Set Enrichment Analysis of differentially expressed genes between Hep3B and PHP (right figure). Core genes is the subset of genes in the geneset contributing to the enrichment signal.
